# Stressors, Resources, and Strain Associated with Digitization Processes of Medical Staff Working in Neurosurgical and Vascular Surgical Hospital Wards: A Multimethod Study

**DOI:** 10.3390/healthcare11141988

**Published:** 2023-07-09

**Authors:** Anika Tell, Joachim Westenhöfer, Volker Harth, Stefanie Mache

**Affiliations:** 1 Institute for Occupational and Maritime Medicine (ZfAM), University Medical Center Hamburg-Eppendorf (UKE), Seewartenstraße 10, 20459 Hamburg, Germany; anika.tell@haw-hamburg.de (A.T.); harth@uke.de (V.H.); 2 Department Health Sciences, Faculty of Life Sciences, University of Applied Sciences (HAW), Ulmenliet 20, 21033 Hamburg, Germany; joachim.westenhoefer@haw-hamburg.de

**Keywords:** digitization, documentation technologies, electronic health records, hospital staff, medical staff, neurosurgery, physicians, preventive measures, technostress, vascular surgery

## Abstract

The digitization of German hospitals is proceeding continuously, leading to the implementation of new digital technologies, such as electronic health records (EHRs) or other technologies, used for the purpose of medical documentation tasks. Even though the replacement of paper documentation through digitized documentation in general promises to come along with plenty of benefits, the daily utilization of technologies might also lead to stresses and strains among the medical staff, eventually possibly leading to the development of different negative work and health-related outcomes. This study, therefore, aims at identifying persisting digitization-associated stressors and resources among medical hospital staff, examining their influences on different work and health-related outcomes, and finally, identifying potential needs for preventive measures. A quantitative study in the form of an online questionnaire survey was conducted among physicians working in the medical field of neuro- and vascular surgery in German hospitals. The study was carried out between June and October 2022 utilizing an online questionnaire based on several standardized scales, such as the technology acceptance model (TAM) and the technostress model, as well as on several scales from the Copenhagen Psychosocial Questionnaire (COPSOQ). The study found medium levels of technostress among the participating physicians (n = 114), as well as low to medium levels of persisting resources. The queried physicians, on average, reported low levels of burnout symptoms, generally described their health status as good, and were mostly satisfied with their job. Despite the prevalence of technostress and the low levels of resources among the surveyed physicians, there is little awareness of the problem of digital stress, and preventive measures have not been widely implemented yet in the clinics, indicating a needs gap and the necessity for the strategic and quality-guided implementation of measures to effectively prevent digital stress from developing.

## 1. Introduction

The digitization and mechanization of work is progressing continuously and has now already reached almost every industry. Everyday work in hospitals is also increasingly characterized by digitization processes, which open new possibilities and opportunities through innovative IT, communication, and device solutions [1]. One of the most current forms of digitization in healthcare is likely to be the introduction of the electronic health record (her), which bundles a variety of health-related patient data, such as information on diagnoses, therapies, or medications. In this way, a transparent and always up-to-date digital patient data overview is offered to medical staff as well as other healthcare professionals [1,2]. According to a recent questionnaire study by Wilhelm et al. (2020), in which German surgeons were asked about their associations with the topic of digitization, EHRs and hospital information systems were the most frequent answers [3]. 

While some countries, such as China, India, the US, and Turkey, and on a European level, also countries such as Denmark, the Netherlands, and Estonia, have taken pioneering positions in hospital digitization [4,5], the state of implementation in Germany, however, is not yet far advanced. According to a global survey conducted as part of the Future Health Index 2019, in which 15 countries were asked about the status of digitization and the utilization of digital technologies in their healthcare systems, digital technologies have so far only been used by 64% of healthcare professionals in Germany. This puts Germany in second last place among the countries surveyed and well below the average usage rate of 78% [4].

According to Stephani et al. (2019), data collected within the framework of the so-called Electronic Medical Record Adoption Model (EMRAM) certification process, which is a method to measure the digitization degree of hospitals based on different criteria and by categorizing the hospitals in different stages (0 = no digitization; 7 = paperless hospital), showed that only 19.2% of German hospitals reached a stage of 5 or higher, while a share of roughly 66% of the hospitals was allocated only to stages 0–2. Furthermore, there currently is no hospital in Germany reaching the digitization stage of 7 [5]. Broken down by the various thematic areas, digital solutions are currently most frequently used in German hospitals in the form of clinical reminder functions (in 43% of German hospitals), followed by support in drug therapy (25%) and applications in medical guidelines and clinical pathways (15%) [6].

It is, by now, commonly known and has already been analyzed in a variety of different studies that the implementation of information and communication technologies (ICT) in hospitals, in general, has a lot of benefits. In a study by Divall et al. (2013), for example, the authors investigated the use of personal digital assistants (PDAs), concluding that PDAs led to an increase in data collection quality and improved the appropriateness of diagnosis and treatment decisions [7]. Further studies have indicated the benefits of ICT, such as an improvement in the traceability of documentation—as patient data can be entered and accessed much easier and faster—or an improvement in work processes and communication between medical and nursing personnel [8,9,10,11]. Especially against the background of the very fragmented structure of care in Germany, the further implementation of EHRs and the further expansion of digital infrastructure, in general, promises to contribute to cost savings potentially caused on a regular basis by a loss of patient data between practitioners, unnecessary double examinations, or uncoordinated treatment processes. Additionally, through the continuing implementation of digital technologies, the transparency, effectiveness, and efficiency of care provision shall be improved [12].

In the medical discipline of surgery, too, there are many possible and promising applications for digital technologies and opportunities for digitizing processes, such as an implementation of virtual outpatient clinics based on the method of telemedicine, for example [13,14], the use or further expansion of the use of surgical robotics or robot-assisted surgeries, which can also be further improved by applying artificial intelligence, machine learning, 5G, big data, cloud technologies, or improved sensors [15], an introduction of hybrid operating rooms with live communication possibilities with external medical experts, or an introduction of electronic medication plans, emergency data management, or digital health applications [15,16]. Especially in the medical discipline of neurosurgery, digital technologies can be highly beneficial, and the neurosurgery sector also has the reputation of being the most digitized medical discipline at the moment. By merging thousands of digital pictures of a tumor-diagnosed patient’s brain, for example, an exact 3D model of the patient’s brain, including the exact location of the tumor, can be developed and utilized during surgery. Along with a creation of a detailed surgical intervention plan, tumor removals like this can be performed very precisely and minimally invasively [17]. Three-dimensional modelling technologies have also made a strong impact in the discipline of vascular surgery. Here, 3D modeling of the vessel to be operated can be of great help for the physicians before and also during the surgical procedure, optimizing the quality of treatment simultaneously [18]. In addition, these discipline-specific technologies can be complemented by further digital technologies used for documentation purposes, such as specific documentation software, which can also be completed with additional functions, such as speech recognition functions to interconnect with recording devices [19]. According to the HIMSS survey (2015) on the topic of digitization and clinical documentation in German hospitals, these additional documentation technology functions are also already in widespread use, especially in the surgery sector, with 11% of the surveyed participants using recording devices with automatic transcript and 3% using devices with the additional speech recognition function [20].

However, despite the above-described benefits, the implementation of ICT and its associated changes might also be accompanied by several disadvantages. In addition to the high costs of the technologies, long-implementation project durations, and aspects of data security, which need to be considered in the implementation process [21], the utilization of ICT also has the potential to lead to stress and strains among the medical staff due to a lack of user-friendliness [22,23].

As of 1 January 2022, the hospitals in Germany were actually obliged by law to fulfil the technical requirements to be able to use EHRs [2]. Against this background and against the background of the introduction of the new Hospital Future Law as well (dt.: Krankenhauszukunftsgesetz (KHZG)), which became effective already on 2 September 2020 and which regulates a big investment program by the government offering fundings for the implementation of ICT in hospitals, and thus, promoting digitization processes, the willingness to invest in and utilize further digital technologies in German hospitals might increase as well. This assumption was reinforced by a recent survey among hospital CEOs in the German federal state of North Rhine-Westphalia, which was conducted in cooperation with the Fraunhofer Institute for Software and Systems Engineering and which showed that indeed most of the clinic managers (with a share of 76%) believed the digitization of the health sector would bring benefits for them, but that the financial resources to implement new digital technologies would not be sufficient [24]. Against this background, it is therefore becoming more and more important to consider the influences of digitization processes in hospitals on the health of the medical staff as well and to gain further knowledge on the interplay of relevant stressors and resources in this context.

### 1.1. Theoretical Framework

It has now already been extensively researched and proven by numerous studies that chronic stress leads to an increased risk for the occurrence of mental and physical illnesses, such as depression, cardiovascular diseases, type 2 diabetes, stroke, cancer, Parkinson’s, epilepsy, and even mortality [22,25,26]. The association of adverse working conditions and the development of adverse health outcomes, in general, has further already been examined in a variety of studies, reviews, and meta-analyses [27,28,29]. To examine and explain the interplay of influencing factors, digital stress, and related outcomes better, the technostress model, the job demands–resources model, and the technology acceptance model were applied in this study and are introduced in the following.

#### 1.1.1. Technostress

Throughout the last decades, the phenomena of digital stress—also known as “technostress”—has gained popularity and is already increasingly researched. Being initially first mentioned by Brod in 1984, who showed that the use of digital technologies in everyday working life does not only come with positive aspects but might also lead to a variety of negative emotions, leading to the development of stress, the so-called technostress model (also the Conceptual Model for Understanding Technostress) was developed by Ragu-Nathan et al. in 2008. Ragu-Nathan et al. defined technostress as “(…) stress experienced by end users in organizations as a result of their use of ICTs” [30]. The model originated from the need for an instrument to assess the exposure to digital stressors as well as the level of persisting technostress and thus obtain a better understanding of the stresses and resource situation of employees at work in general. Therefore, Ragu-Nathan and his research team developed and empirically validated a questionnaire consisting of different items belonging to two main constructs: the “technostress creators”, or digitization-related stress factors, and the “technostress inhibitors”, which correspond to digitization-related resources or protective factors [30].

Following the performance of exploratory and confirmatory factor analyses, the researchers identified the following five factors within the construct of technostress creators: “techno-overload”, “techno-invasion”, “techno-complexity”, “techno-insecurity”, and “techno-uncertainty”. While the factor of techno-overload refers to work situations where the employees are forced by the digital technologies to work longer and faster, techno-invasion refers to situations where the utilization of digital technologies make the employees feel they can be reached at all time, which ultimately leads to a blurring of work and private life [30]. The factor of techno-complexity further describes the fact that employees might feel inadequate regarding their technology skills due to the complexity of the technologies, whereas the factor of techno-insecurity refers to work situations where employees might fear the loss of their job due to the continuously proceeding automatization of work processes or due to better skilled fellow colleagues. Additionally, the factor of techno-uncertainty describes situations where employees worry and feel uncertain due to the progressing changes in technology [30].

Furthermore, the authors identified the technostress inhibitor factors, including “literacy facilitation”, “technical support provision”, “involvement facilitation”, “job satisfaction”, “organizational commitment”, and “continuance commitment” [30]. While the inhibitor of literacy facilitation refers to the encouraging and fostering of the sharing of ICT-related information and knowledge, the inhibitor of technical support provision means that measures are provided to solve ICT-related problems and to help the employees in their application of the ICT, thus reducing technostress. The inhibitor of involvement facilitation further describes the fact that employees are currently updated on ICT-related changes and the reasons for it [30]. The technostress inhibitors of job satisfaction, organizational commitment, and continuance commitment further describe the satisfaction of employees with their job, their employer, and the lack of need to change their employer [30].

The developed technostress instrument by Ragu-Nathan et al. (2008) and its items have since been utilized in a variety of different studies aiming to assess the technostress level of employees working in a variety of different branches and work settings and measuring different outcomes [30,31,32,33,34,35,36,37,38,39,40,41].

#### 1.1.2. Job Demands–Resources Model

In a more general context, the research on technostress can also be complemented by other models explaining the interplay of stressors and resources at a work organizational level in general, such as by the job demands–resources model by Demerouti et al. (2001) [42]. This well-known model describes the interplay of characteristics of the work tasks and of the work environment, and from it, the resulting demands and resources, and eventually, the effects they might have on the employees. In detail, the JD-R model states that both demands and resources can persist on many different levels.

Furthermore, it is important to note that job demands are not necessarily negative and only develop into job stressors once the effort to fulfil them is too high or the influence of persisting job resources is too low. This underlines, once again, the importance of the resources and the interplay of demands and resources, which, in the following, then decide on the emergence of stress and negative outcomes or of well-being and positive outcomes [43].

#### 1.1.3. Technology Acceptance Model

Furthermore, the usage perception, and therefore, the attitude a person has towards using a digital technology, plays a role in the development of stress reactions associated with the utilization of digital technologies, too. A qualitative study by Körner et al. (2019), for example, found that the level of the perceived stress resulting from human–machine interactions depended, among others, on the usability of a technology [44]. Virone et al. (2021) further showed in their systematic literature review that the aspect of the design of a technology, corresponding to the perception of the technology, and consequently, to the classification as user-friendly or not, was significantly associated with the level of technostress [45].

One way to measure the usage perception and acceptance of digital technologies, and thus, to also make assumptions about the levels of digital stress, is with the TAM. This model, which is commonly considered the gold standard of instruments to predict technology acceptance, was developed in 1989 by Davis, and is based on two well-established psychological models of behavior, more specifically the theory of planned behavior (TPB) and the theory of reasoned action (TRA) [46,47,48]. The model consists of the two main determinants of “perceived usefulness” (PU) and “perceived ease of use” (PEOU), which, briefly explained, have mediating roles between the external variables, such as the characteristics of the respective technology and the attitude of the user towards the technology and their behavioral intention to use it, which then eventually has an influence on the actual utilization of the technology [47]. Whereby the determinant of perceived usefulness, according to Davis, corresponds to the subjective perception of the usefulness of a technology for doing what the end user wants to do, the determinant of the perceived ease of use corresponds to the degree to which a person believes that using the respective technology would be easy [47].

Since its first introduction, the model has been further developed into the two additional model versions of TAM 2 and TAM 3 and has been used to form the basis—among other models as well—for the unified theory of acceptance and use of technology (UTAUT) [47]. Despite, of course, some differing characteristics of the model extensions compared to the initial model, the two determinants of PU and PEOU remained as strong influencing factors in all model versions.

### 1.2. The Digitization-Associated Stressor and Strain Situation of Medical Staff

There is a solid study base that medical and nursing hospital staff, in general, are exposed to a broad variety of psychological stress factors, which have the potential to lead to adverse health outcomes and even to the manifestation of mental health disorders. In most of the most recent studies, the level of adverse mental health outcomes and psychological disorders has been measured under the influence of the COVID-19-pandemic, most of them specifically focusing on pandemic-related stressors. According to the study by Sangrà et al. (2021), for example, almost 13% of the surveyed healthcare workers experienced severe anxiety, 26% reported high levels of perceived stress, more than 23% showed severe symptoms of posttraumatic stress, and about 13% of the employees reached scores high enough to be compatible with the diagnosis of a major depressive disorder (MDD) [49].

Older studies, leaving out the influence of pandemic-related stressors, however, still showed high levels of mental disorders. According to an overview paper by Grundmann et al. (2012), burnout rates ranged between 16 and 30% among nursing staff and between 13 and 28% among physicians working in hospitals. The rates of the single burnout dimensions were sometimes even higher, especially among clinical and surgical oncologists, with the levels of the dimension of “emotional exhaustion” from 22% to 53.3%, the rates of the burnout dimension of “depersonalization” between 7.4 and 30%, and the levels of “personal accomplishment” between 14.2% and 69% [50]. According to another more recent study by Shanafelt et al. (2015), burnout levels among physicians, in general, also seem to have risen in recent years [51].

It has already been shown in several studies that digital technologies have not always led to a workload reduction in the medical field, particularly in hospitals, but other than promised, have often even had the contrary effect, leading to increased administrative burdens, for example, specifically in the case of electronic health records [52]. A recent study by Wong (2020), for example, showed that “(…) for every hour physicians spent on direct patient care during the clinic day, they spent an additional 2 h on electronic health records” [53]. According to Shanafelt et al. (2016), physicians are often not satisfied with the amount of time spent using electronic health records or computerized physician order entry and are at higher risk for experiencing burnout [54].

In other studies, for example, such as those by Vehko et al. (2019) and Yan et al. (2021), in which nurses were asked about their perception of EHRs, most of the study participants reported that they were stressed by EHRs. They specifically pointed out that they experienced high time pressure since the implementation of EHRs, perceived the technology as unreliable and not user-friendly, and associated the technology with a higher workload and a reduced amount of quality time spent with patients [55,56].

In another—and also very recent—qualitative study, which was carried out among nursing leaders, additional stressors were identified by the research team, such as insufficient education or training, no inclusion in the development processes of technologies, a lack of time to learn, bad user interfaces or functionalities of technologies, too many different technologies or digital services, or too fast changes and implementations of technologies [57]. These findings are complemented by the study results of Stadin et al. (2020), who identified three main areas of digitization- or technology-associated stressors. These areas were the negative aspects of digital communication in general, such as a high workload or negative feelings, a poor user experience of ICT systems, including technical struggles or illogical ICT systems, and lastly, the need to improve organizational resources, including a lack of general digital literacy, and consequently, the need for practical training or the need for increased administrative support [58]. The research team around Laukka (2023) also identified some potentially important influencing resources. These were, for example, information and training, a positive attitude towards technologies, self-efficacy, knowledge transfer and transparency regarding the digital strategy of the hospital, perceiving the technologies as beneficial, having positive role models, and knowledge about role model effects within the hospital [57]. These findings are also complemented by the findings of Stadin et al. (2020) again, who additionally pointed out culture, norms and social support (e.g., situation-based coworker support), individual resources (e.g., digital literacy, confident attitude), and organizational resources (good IT support, administrative support, back-up routines, etc.) as important resources. The need for improvement were further identified regarding digital literacy facilitation, user influence, and a redistribution of work and ICT systems [58].

At this point, it is also important to point out that especially one’s attitude toward technology influences the degree of technology-related stress experienced by the individual [59,60,61]. According to the research team, digitization-related stress can be differentiated into techno-distress and techno-eustress, meaning that the actual technostress outcome essentially depends on the appraisal of the technology [59].

In another very recent study among German healthcare workers (mainly physicians), the research team around Gaube (2021) proved that a good or positive perception of technologies was associated with lower techno-overload scores and with lower IT-related stress in general. Higher techno-overload scores and higher digitization-associated stress were also associated with lower job satisfaction [62].

#### Current State of Research

In a recent study by Gardner et al. (2019), the authors showed that 70% of the physicians who utilized EHRs on a regular basis experienced digital stress and that physicians who reported a moderate to an excessive amount of time spent on EHRs at home had odds 1.9 times higher for developing burnout [63]. These results are corroborated by another recent study by Golz et al. (2021), in which the digital stress levels of healthcare professionals working in psychiatric hospitals were measured. The study was able to show moderate stress levels among the formerly named study group, whereas nurses and physicians reported slightly higher stress levels than other professional groups working in the hospitals, such as social workers and psychologists, for example [38]. In another study by Tajirian et al. (2020), 69.3% of the queried physicians also stated that they felt like EHRs always or almost always contributed to their burnout symptoms [64]. Technostress levels—the same as burnout rates— also varied among different medical disciplines, whereby physicians working in the field of orthopedic surgery were most frequently affected, with up to 86.5% of the queried physicians reporting digital stress, while digitization-associated stress levels among pediatricians, for example, only affected 33.6% [63]. Golz et al. (2021), in addition, also examined the influence of technostress levels on several health- and work-related outcomes and identified technostress as a relevant predictor for the outcomes of burnout symptoms, job satisfaction, intention to leave the profession, intention to leave the organization, general health status, quality of sleep, headaches, and work ability (the latter, however, was least impacted) [38]. Higher social support, on the other hand, seemed to decrease technostress and was, therefore, significantly negatively associated with it [65].

Furthermore, the variables of age and gender seem to have an influence on the expression of technostress and the outcomes of burnout symptoms. The general study situation on digital stress indicates that stress levels are higher for female study participants and for participants of younger age [66]. However, when comparing these results with the results from studies measuring digital stress among physicians, Golz et al. (2021), for example, showed higher technostress levels for older healthcare professionals [38]. Gardner et al. (2019) further found that female gender was associated with higher odds of having burnout symptoms among the queried group of physicians [63]. Female physicians and younger physicians were also identified to have higher odds of developing burnout symptoms in several other studies [67,68,69].

According to the HIMSS Europe study, especially the time spent on digital documentation tasks appeared to have an influence on job satisfaction and decreased it [20].

Esmaeilzadeh et al. (2021) further identified specific EHR-related stress factors, which were, e.g., inadequate training for using the technology, having less face-to-face time with patients, spending too much time on data entry, and increasing computerization at work in general [23]. According to Tajirian et al. (2020), especially the physicians that showed higher levels of frustration with EHRs and were less satisfied with them in general experienced more burnout symptoms than other physicians [64]. However, it was indicated that a positive perception of use was associated with lower odds of burnout symptoms among healthcare personnel [23].

In summary, studies investigating digitization-associated stressors and resources as well as health- and work-related outcomes among German hospital staff are still rare at the moment. As far as we are concerned, there are currently no studies focusing solely on the professional group of physicians, and in addition, none focusing specifically on the different professional levels, such as assistant physicians. While the technostress levels of medical staff working in specific medical professions already have been sporadically explored, and comparisons among certain medical professions already have been conducted in some studies, there are, at the moment, no studies assessing technostress levels and the relevant outcomes in the medical professions of neurosurgery and vascular surgery. In addition, there are no studies examining the need for and the current status of the implementation of measures preventing technostress and stress-related outcomes.

However, the current scientific evidence on technostress, in general (without a limited focus on medical staff or surgeons), has already showed high stress levels of employees working with digital technologies and has already identified a variety of technostress creators as well as protective factors. The above-described studies on the persistence of technostress in the health sector additionally already give a first impression about the prevalence of technostress among medical staff and about persisting digitization-associated stressors, resources, and outcomes. However, the research on digital stress in hospitals and especially among the group of physicians working in surgery departments is not yet far advanced, and research in this field is rare, and therefore, highly indicated.

### 1.3. Objectives

The aim of this study was to conduct a quantitative study (1) to analyze the associations among digitization processes and the daily work of medical staff working in neurosurgery and vascular surgery clinics, (2) to determine the stressors and resources evolving from the utilization of digital technologies, and (3) to examine the handling of and coping with digital stress. Furthermore, (4) the relationships among relevant health- and work- related outcomes, and (5) the need for preventive measures was examined, and thus, a contribution to closing the existing data gap is made. Against this background, the following hypotheses were formulated:

**H_1_.**  *Lower ratings of the subjectively perceived usefulness and the perceived ease of use of the utilized digital technologies are associated with higher expressions of techno-stressors or higher technostress levels of the medical staff working in the named chirurgical wards.*

**H_2_.**  *Higher expressions of the technostress creators or higher technostress levels among the medical staff working in the named chirurgical wards are (a) associated positively with higher rates of burnout symptoms. (b) associated negatively with lower levels of job satisfaction. (c) associated negatively with a lower-rated general health status.*

**H_3_.**  *A higher expression of the technostress inhibitors or resources among the medical staff working in the named chirurgical wards is (a) associated negatively with lower rates of burnout symptoms. (b) associated positively with higher levels of job satisfaction. (c) associated positively with a higher-rated general health status.*

**H_4_.**  *There are significant differences in the expressions of techno-stressors or technostress levels, and consequently, the rates of burnout symptoms (a) among the different age groups. (b) between men and women.*

**H_5_.**  *Technostress levels among medical staff working in the named chirurgical departments are significantly lower in those employees whose employers have already implemented preventive measures, such as the provision of information or qualifications, to a higher degree.*

## 2. Materials and Methods

### 2.1. Study Design and Study Population

This study was conducted in the form of a quantitative, cross-sectional, online-based questionnaire survey in specialized neurological and vascular chirurgical clinics in Germany. The eligibility criteria for the study participants was defined as having an occupation as a physician in a specialized neurosurgery or vascular surgery clinic, either as a head physician, senior physician, specialist physician, or as an assistant physician. As a secondary criterion, it was defined that the study participants must utilize digital technologies for clinical documentation purposes, such as EHRs or other documentation software or additional hardware/digital devices, e.g., for medical basic documentation or hygiene documentation, etc., at least once a week, consequently meaning, of course, that the chirurgical wards must have implemented at least one of such digital technologies or group of technologies. As the eligibility criteria for the hospitals, it was defined that the hospitals should either have a whole specialized neurosurgery or vascular surgery clinic or at least a separated section for the chirurgical discipline, but the size of the clinic did not play a role in the selection of the hospitals. However, chirurgical practices, polyclinics, and outpatient clinics were not included.

A minimum total sample size of n = 200 was targeted, calculated using G*Power version 3.1.9.6 and by assuming an a priori power analysis with an alpha = 0.05, 95% confidence intervals, and a medium effect size for all planned analyses. The two medical disciplines of neurosurgery and vascular surgery were chosen because digital documentation technologies are already rather widespread in the surgery sector compared to other medical disciplines, and thus, could ensure the achievement of the targeted sample size.

### 2.2. Data Collection

The online survey was conducted within a period of roughly two months from the end of June to mid-October 2022 using the survey platform Lamapoll. The corresponding relevant neuro- and vascular surgical clinicals were identified via registers and lists from the website pages of the two respective medical societies of the German Society of Surgery. The registers were further supplemented with the results of the German hospital search portals “Weisse Liste” (Engl.: White List) and “Deutsches Krankenhausverzeichnis” (Engl.: German Hospital Directory). In total, 403 hospitals were included in the study acquisition, including 185 neurosurgery wards and 352 vascular surgery wards. The study participants were then initially recruited via E-mail contact with the leading physicians or the secretaries of the chirurgical clinics, providing detailed information about the research project, the research aims, and the survey link as well as an information flyer with further information about the study project for distribution within the clinic. In cases where the E-mail addresses were not provided on the hospital websites, the Society of Surgery website, or other websites, the clinics were first contacted directly via telephone. In summary, the study invitations were sent to a pool of approximately 4154 physicians (physicians working in neurosurgery clinics: 1720; physicians working in vascular surgery clinics: 2434).

After 3 weeks, reminders were sent to all clinics. Furthermore, some additional clinics were identified in further hospital search portals (Klinikradar, Kliniken.de) and recruited according to the above-explained approach.

Prior to the start of the actual data collection, the thematic fit and the comprehension of the utilized items, as well as the scope of the whole questionnaire, were checked in a pre-test by a total of 12 scientific experts. The feedback and the different resulting suggestions for improvement were then discussed within the research project group, and the questionnaire was revised accordingly.

### 2.3. Measures

The online survey consisted of seven thematic blocks. Prior to the start of the actual online survey, the study participants were first provided, once again on the starting page, with more detailed information about the research project, the research aims, and the applicable data protection standard, and they were informed that their participation in the study was voluntary.

#### 2.3.1. Sociodemographic and Work-Related Variables

In the first block of the questionnaire, sociodemographic data were queried first to collect necessary information on the participants’ chirurgical discipline, job position/career status, utilization of digital documentation technologies, age, sex, regional structure and ownership of the clinic, duration of occupation in the respective chirurgical clinic, and work experience in the field in general, as well as weekly working hours.

#### 2.3.2. Techno-Stressors and Technology-Associated Resources

In the second thematic block, the persistence of digital stressors in the workplace was measured. For this, the standardized and validated technostress scale by Ragu-Nathan et al. (2008) was used in an adapted version, including the three technostress creators of techno-overload, techno-complexity, and techno-uncertainty in the German version, with a total of 14 items [30]. This instrument has acceptable to good reliability with Cronbach alpha values for the different constructs ranging between 0.71 and 0.87 and good discriminant and convergent validities, with no significant error correlations between the items [30]. Additionally, for a more specific query of the stressors, another self-developed item was utilized based on an item from the HIMSS study (2015) [20]. To obtain an overview about the persisting protective factors or resources in the working place, in the next block, the two technostress inhibitor constructs of literacy facilitation and involvement facilitation from the already mentioned technostress scale by Ragu-Nathan et al. (2008) were used, with a total of 9 items [30].

#### 2.3.3. Preventive Measures

The third thematic block covered several items for the query of preventive measures with two constructs, of which the one by Bräutigam et al. (2017) was slightly changed, assessing the already implemented preventive measures with Likert-scale (8 items in total) [70,71,72]. These items were further complemented by a self-developed scale querying the benefit of the already implemented preventive measures as well as by three additional self-developed items in free-text format intended to capture the positive and negative aspects of the preventive measures as well as the need for further preventive measures.

#### 2.3.4. Work- and Health-Related Outcomes

Next, several health- and work-related outcomes were assessed in another thematic block, all by utilizing standardized and validated scales. The outcome of burnout symptoms was measured with the homonymous standardized and validated scale from the COPSOQ 2022, which consisted of 3 items. Furthermore, the outcome of job satisfaction was measured with the 3-item-construct by Ragu-Nathan et al. (2008), and for the outcome of general subjective health status, a 10-stepped scale (0 = lowest level of health, 10 = highest level of health) from the COPSOQ 2022 was used [30,72].

In the analysis, the COPSOQ-scale of burnout symptoms showed good reliability, with a Cronbach’s alpha value of 0.84. As the general subjective health status scale only consisted of one item, a reliability value was not calculated [73]. For the utilized job satisfaction scale, the reliability was also shown to be high, with a Cronbach’s alpha value of 0.87 [30].

#### 2.3.5. Usage Frequency and Attitudes Regarding Digital Technologies

Lastly in this questionnaire, the frequency and duration of the utilization of digital documentation technologies as well as the participants’ attitude towards the technologies were measured. For the assessment of utilization frequency and duration, two self-developed items were utilized, with the one measuring the utilization frequency based on the studies by Hübner et al. (2018) and Gensicke et al. (2016), and the one measuring the duration of use based on the HIMSS study (2015) [20,74,75]. Additionally, for the query of the attitudes towards the utilized technologies, the two validated construct scales of perceived usefulness (PU) and perceived ease of use (PEOU) from the German version of the technology acceptance model (TAM) were used [46]. Cronbach’s alpha values were at 0.875 for the PU scale and at 0.806 for the PEOU scale, thus indicating good to almost very good reliability [46]. For more details regarding the utilized scales and items, please find the questionnaire in the annex.

### 2.4. Statistical Data Analysis

For the evaluation of the data, descriptive and analytical analyses were conducted using IBM SPSS version 27 statistical software. Initially, the data were checked for plausibility, revealing no suspicious cases. The data were checked for missing values, and plausibility and frequency distributions were generated prior to the actual analyses. Then, 95% confidence intervals and an α-level of ≤0.05 were set for the significance tests. The descriptive statistical data analysis included frequency distributions (absolute and relative frequencies as well as the relevant location parameters, such as the median, mean, standard deviation, range, and quartiles).

For the testing of the first, second, and third hypotheses, correlation analyses (Pearson’s correlation coefficient for continuous variables, Spearman’s Rho correlation coefficient for ordinal variables) were conducted. Fulfilment of the necessary requirements for conducting Pearson’s correlation analyses was checked in a first step through the generation of graphs with a normal distribution curve, boxplots, QQ plots, scatterplots, skewness and kurtosis values, and tests for normal distribution. If the requirement for the normal distribution of the continuous variables was not fulfilled, the Pearson correlation analyses were conducted applying the bootstrapping method.

For the statistical analysis of group differences, non-parametric tests (Chi^2^ test, Mann–Whitney U-test, Kruskal–Wallis test) were carried out for the categorical variables and for continuous variables not fulfilling the prerequisites of parametric tests. For the continuous variables, appropriate parametric test procedures (*t*-test, ANOVA) were applied after testing for the normal distribution of the variable data. For the statistical testing of correlation hypotheses (Hypotheses 1–3), regression analyses were applied, controlling for potential confounding variables. Once again, if the requirements for the respective analyses were violated, the analyses were conducted by applying the method of bootstrapping.

## 3. Results

### 3.1. Sample Description

A total of n = 168 physicians working in either neurological or vascular surgery clinics participated in the online survey. After checking for missing values and plausibility, 54 questionnaires had to be excluded, and consequently, were not included in any statistical data analysis. Among the remaining 114 study participants, a total of 52 physicians worked in neurosurgery clinics, whereas 62 physicians worked in vascular surgery clinics. Most of the participating physicians were male (68.4%; n = 114) and belonged to the age group of 50–59 years (32.5%; n = 114) (see Table 1 for details).

Furthermore, most of the physicians were employed as senior physicians (46.5%*;* n = 114), had been employed in their clinic for more than 4 years (76.3%; n = 114), and in general, mostly had been working in the clinical field for more than 25 years already (19.3%; n = 114) (see Table 1). Regarding the usage of digital documentation technologies, the electronic health record (EHR) was the most frequently chosen answer category (87%; n = 114), followed by the options of additional software (83%) and additional digital devices or hardware (65.8%).

### 3.2. Descriptive Statistical Analysis

#### 3.2.1. Usage Frequency and Usage Duration of Digital Documentation Technologies

Regarding the usage frequency of the digital documentation technologies, almost all participating physicians indicated they would use the technologies on a daily basis (99.1%; n = 114) (for further details, see Table 2).

#### 3.2.2. Techno-Stressors and Stress-Inducing Aspects of Documentation Technologies

In general, the measured average technostress level of all participants was at a medium level, with a mean of the three technostress-creators of M = 2.8 (1 = do not agree at all/no technostress; 5 = fully agree/high technostress levels) and an SD = 0.65. Regarding the single technostress creators, the highest mean was observed for the construct of techno-overload (M = 3.09; SD = 0.86) (see Table 3).

As described in detail in Section 2, several potential negative side effects or stress-inducing aspects were queried in addition. With a share of 18.4% (n = 114), the aspect of double documentation was the most frequently perceived as stress-inducing, followed by the aspects of technical errors of the system, with a share of 15.8%, control tool of the health insurances (14.9% of physicians), and lack of PC workstations (14% of physicians). Regarding the aspect of double documentation, more than half of the participants stated to be stressed by this side effect of the technologies frequently or very frequently. The aspect of lack of data security did not seem to be perceived as a problem, as 62% of the participants stated being either never or only very rarely stressed by it.

#### 3.2.3. Technostress Inhibitors/Resources

The mean of the overall expression of technostress inhibitors or resources was assessed with M = 2.51 (SD = 0.85) corresponding only to a low to moderate level of persisting resources. While the technostress inhibitor of literacy facilitation was at a moderate level (M = 3.1; SD = 1.05), the level of the inhibitor of involvement facilitation was especially low, with a mean of only M = 1.91 (SD = 0.9), meaning that this inhibitor was not perceived as a strong resource (see Table 4 below).

#### 3.2.4. Status Quo and Benefits of Implemented Preventive Measures

Overall, on average, preventive measures were implemented rarely or only to a moderate degree (M = 2.51; SD = 0.78). The most frequently implemented preventive measures were qualifications, with the physicians stating with a mean of 2.98 (SD = 1.06) that they received an additional qualification if needed or that they were sufficiently qualified in the course of the introduction of new technologies. In addition, 76% of the participants (n = 114) stated that their employer would provide a sufficient number of end devices. However, only 26% of the participants declared that their employer considered introducing end devices that would not interfere with the conversation between the doctor and patient. Also, only 21% of the physicians stated their employer planned the technology rollout only after ensuring the stability of the system to reduce time-consuming double documentation. Regarding the benefit of the already implemented preventive measures, almost 70% (n = 114) of the physicians were at least satisfied with the measures. However, only 5.3% of the participants were very satisfied, and in addition, over 30% of the physicians also stated they were not satisfied or were not satisfied at all with the implemented preventive measures.

#### 3.2.5. Technology Acceptance

The assessment of technology acceptance with the TAM model resulted in a mean of M = 3.42 (SD = 0.82, n = 114) for the construct of perceived usefulness, meaning that the physicians, on average, agreed with the items or were neutral, corresponding to a rather useful perceived view on the utilized technologies. Regarding the construct scale of perceived ease of use, the query resulted in an overall mean of M = 3.05 (SD = 0.67, n = 114), slightly lower compared to the first construct scale, meaning that the average physician neither perceived the utilized technologies as easy to use nor as not easy to use.

#### 3.2.6. Work- and Health-Related Outcomes

Overall, the persistence of burnout symptoms among the physicians, on average, was at a low level (M = 2.92; SD = 0.97; n = 114), correspondingly meaning that the average physician did experience burnout symptoms only randomly from time to time (see Table 5 below). Regarding the subjectively perceived general health status, the participants (n = 114), on average, perceived their health as good, with a mean of 7.62. The SD however, with a value of 1.84, was rather high, and the answers, in addition, also ranged from the scale expression of 1 to 10. Lastly, on average, most of the physicians were satisfied with their job, with a mean of 4.05 (SD = 0.63) (see Table 5).

### 3.3. Qualitative Data Analysis

In addition to the already described quantitative findings, the study participants gave further insights into which aspects of digital documentation technologies they perceived as stress-inducing and provided further information about their subjective perception of measures, aiming at preventing technostress. In total, 4 main categories and 24 subcategories were formed (see overview in Table 6), which are described in more detail in the following.

#### 3.3.1. Stress-Inducing Aspects of Digital Documentation Technologies

In the first step, the participating physicians were asked about their view on stress-inducing aspects of the (newly) introduced technologies. A total of 26 physicians (n = 114) answered this question. Stressors mostly originated from the aspects of technology itself as well as from organizational and human factors. Specifically, regularly occurring technical failures and immature technology solutions, in general, were perceived as stress-inducing. This stressor was also connected to the fear of being too dependent on the technologies, as technical failures might have very serious consequences. In addition, a lack of support from the IT team as well as planning deficits during the rollout phases of the new technologies were described as stressful, as they often led to longer working hours and an increased workload due to double digital and analog documentation, for example.

*“Here, systems are installed without prior consultation and we are then supposed to test them. This is thought to save money. However, the time required is significantly higher and such behavior is not worthwhile in the long term”*. (P81; Engl. translation of original citation.)

Furthermore, a skeptical position of the hospital management regarding the introduction of new technologies, as well as unclearly defined usage purposes and insufficient adaptions of the technologies to the daily clinical practice were perceived as stressful.

*“Problem in my eyes: The development/acquisition of new IT systems always has the focus on controlling/administration, the daily MEDICAL aspect is mapped far too little. The result is absolutely unintuitive systems with multiple analysis functions that hinder rather than promote the daily application process”*. (P44; Engl. translation of original citation.)

#### 3.3.2. Benefits of Implemented Preventive Measures

It was next especially of interest to the research team to gain further insights into the usage perception of the preventive measures to reduce technostress. A total of 30 physicians (n = 114) gave answers on this item. As the first aspect, the provision of up-to-date information and transparency during the induction phase of new technologies was seen as beneficial. A high quality of technology and system maintenance service was perceived as helpful, too. Furthermore, it was perceived as essential to involve and consult with the physicians already in the selection phase of new technologies and to plan realistic technology roll-outs oriented towards the special characteristics of daily working life in clinics.

*“As a head physician, you have a very good influence on developments, this does not apply to the same extent for subordinate levels”*. (P65; Engl. translation of original citation.)

In addition, an adaption of the preventive measures, such as trainings, to the needs of the clinic personnel was perceived as beneficial in order to ensure the possibility of participation, for example.

*“Training courses are offered but often cannot be attended in everyday life due to lack of time”*. (P104; Engl. Translation of original citation.)

In addition to the above-mentioned, there were also a lot of physicians stating that preventive measures had not been implemented at their clinics yet at all.

*“Preventive measures, as mentioned above, do not take place to any significant extent”*. (P82; Engl. translation of original citation.)

#### 3.3.3. Criticism Regarding Implemented Preventive Measures

Being asked about the not-so-beneficial aspects of the implemented preventive measures, a total of 21 participants (n = 114) gave some insights. In congruence with the previously described aspects, specifically a lack of involvement in the technology selection processes, a lack of support from the IT team during the introduction phase, and a lack of time to participate in trainings or a lack of adaption of trainings to clinical daily routines were perceived as negative.

*“There is no involvement of end users in decision-making processes, and digitization is years behind competitors or the requirements of the KhZG in terms of implementation. Familiarization with existing software is largely left to the users themselves”*. (P82; Engl. Translation of original citation.)

In addition, the physicians mentioned that technology trainings were either not existing at all or were too short to ensure successfully working with the new technologies, and therefore, were not effective.

#### 3.3.4. Wish for Additional Preventive Measures

Furthermore, when asked if they wished for additional measures to prevent stress induced by the usage of digital documentation technologies, a total of 24 physicians (n = 114) answered. As one main point, the participants wished for regular involvement in the technology selection processes and in the design and planning of introduction processes of new technologies. Regarding the planning and conduction of technology trainings, the participants, on the one hand, wished for additional comprehensive information, training and support from the IT team, and on the other hand, wished for a simplification of the training contents.

*“Involvement in decision-making processes, familiarization with new software and technology, availability and competent support from the IT department”*. (P82; Engl. translation of original citation.)

Furthermore, they commented again that the trainings should be adapted to the needs of the clinic personnel and to everyday clinic routines. As a last point, the physicians added they would like to be informed about best practice—examples from other clinics—and to learn from other clinics, too.

*“One could also buy systems/software whose functional benefit has been proven elsewhere”*. (P11; Engl. translation of original citation.)

### 3.4. Analytical Statistical Analysis

The analyses to test our first hypothesis showed a moderate negative correlation between the variables of subjectively perceived usefulness (PU) and technostress, r = −0.372 (CI: −0.529, −0.225), *p* < 0.001, as well as a moderate negative correlation between the subjectively perceived ease of use (PEOU) and technostress, r = −0.452 (CI: −0.610, −0.275), *p* < 0.001 (for further details see Table 7 below). Consequently, these findings support Hypothesis H_1_.

The multiple regression analysis resulted in an adjusted R-squared value of 0.252, meaning that 25% of the variance of the dependent variable could be explained by the two independent variables of subjectively perceived usefulness (PU) and subjectively perceived ease of use (PEOU), respectively, by this model. The analysis result for the whole model was highly significant (*p* < 0.001). The single influence of the two independent variables was significant too. For the variable of perceived usefulness, the coefficient value was b = −0.183 (CI: −0.318 to −0.048), *p* < 0.05, and for the variable of perceived ease of use, the coefficient value was b = −0.311 (CI: −0.478 to −0.144), *p* < 0.001, meaning that with each increase in the variables of PU and PEOU of the respective digital technologies, the technostress levels of the employees decreased (see Table 8 below).

In support of Hypothesis H_2a,c_, the Pearson correlation analyses resulted in a small positive correlation between the variables of technostress and burnout symptoms, r = 0.214 (CI: 0.008, 0.395), *p* < 0.05, and in a small negative correlation, r = −0.194 (CI: −0.411, 0.027) with *p* < 0.05 for the variables of technostress and general health status. For the variables of technostress and job satisfaction, we found a small negative correlation too, with r = −0.182 (CI: −0.4, 0.04), which, however, was not significant (*p* > 0.05) (see Table 9 below). Thus, we had to reject Hypothesis H_2b_.

The multiple regression analysis for the techno-stressor-variables and the outcome burnout resulted in an R-squared value of 0.125, meaning that 12% of the variance of the dependent variable burnout could be explained by the three independent variables of techno-overload, techno-complexity, and techno-uncertainty, respectively, by this model. The results were also highly significant (*p* < 0.001). The influence of the predictor of techno-overload was highly significant (*p* < 0.001), with a coefficient value of b = 0.442 (CI: 0.242, 0.641), meaning that with each increase in the unit of techno-overload, the burnout scores of the physicians increased as well. The regression results of the two other predictors, however, were not significant (*p* > 0.05) (see Table 10).

The adjusted R-squared value for the multiple regression analysis of the techno-stressor variables and the outcome job satisfaction was 0.068, meaning that a rounded 7% of the variance of the dependent variable of job satisfaction could be explained by the three independent variables of techno-overload, techno-complexity, and techno-uncertainty by this model. The result was significant too (*p* < 0.05). However, only the influence of the predictor of techno-overload was significant (*p* < 0.05), with a coefficient value of b = −0.163 (CI: −0.297, −0.029), meaning that with each increase in the unit of the variable of techno-overload, the dependent variable job satisfaction decreased by a unit. The regression results of the other two predictors, however, were not significant (*p* > 0.05) (see Table 10 below).

For Hypothesis H_3_, the correlation analyses of the two technostress inhibitor variables and the burnout variable were not significant, with *p* > 0.05, nor were the analyses for the technostress inhibitors and the variable of general health status, which resulted in a small positive, r = 0.118, yet also not significant result, with *p* > 0.05. Thus, we had to reject Hypotheses H_3a_ and H_3c_. However, in support of Hypothesis H_3b_, we found a small positive correlation for the two technostress inhibitor variables and the outcome of job satisfaction, with r = 0.190 (0.009, 0.355) and *p* < 0.05 (see Table 11 below).

We next conducted a one-way ANOVA to assess the influence of age on technostress levels. Age was divided into five groups: 20–29 years (*M* = 2.85, SD = 0.77), 30–39 years (M = 2.55, SD = 0.63), 40–49 years (M = 2.70, SD = 0.64), 50–59 years (M = 3.00, SD = 0.64), and 60 years or older (M = 2.89, SD = 0.68). The level of technostress differed statistically significant among the different age groups, with *F* (df: 4, 139) = 2.667, *p* < 0.05. The eta-squared values (n^2^) were 4.504 (between groups) and 63.194 (total) = 0.07, corresponding to a medium Cohen effect size [76,77].

We then further conducted a Kruskal–Wallis-analysis to identify if the expressions of burnout symptoms differed among the different age groups. The results, however, were not significant, consequently meaning that there were no differences regarding the expression of burnout symptoms between the different age groups in our data set. Our findings, therefore, do not fully support Hypothesis H_4a_.

To identify if differences regarding the expression of technostress levels persisted between the genders, we then conducted a t-test for independent samples. The results of the analysis were not significant (*p* > 0.05), with t-value: 0.65, df: 142, consequently meaning that there was no difference regarding the expression of technostress between men and women.

We lastly conducted a Mann–Whitney-U-test to check for differences regarding the expression of burnout symptoms between men and women. The test results show a statistically significant difference in the expression of burnout symptoms between the two genders, consequently supporting Hypothesis H_4b_ in part. Female study participants showed higher rates of burnout symptoms (M = 84.11) than male study participants (M = 61.53), with the exact results of the Mann–Whitney U-Test: *Z* = −3.104, *p* = 0.002. The effect size according to Cohen (1988), however, was r = 0.02, corresponding only to a small effect [76,77]. 

To check our last hypothesis, we conducted a one-way ANOVA for the above-described variables of preventive measures and technostress. The variable of preventive measures was divided into the following three groups: “do not agree” (corresponding to a low level of implemented preventive measures; M = 2.91, SD = 0.71), “partly agree” (corresponding to a medium level of implemented preventive measures; M = 2.77, SD = 0.63), and “agree” (corresponding to a high level of implemented preventive measures; M = 2.38, SD = 0.40).

The levels of technostress differed statistically significantly among the different groups of implementation of preventive measures, with *F* (df: 2, 135) = 2.093, *p* < 0.05. The eta-squared values (n^2^) were 4.186 (between groups) and56.525 (total) = 0.07, corresponding to a medium effect according to the rules of Cohen. This result is in support of Hypothesis H_5_. When checking the post-hoc analysis results to learn which groups exactly differed regarding the Technostress levels, the Games–Howell test showed significant differences between the groups of “do not agree” and “agree”, with a mean difference of 0.53 (*p* < 0.001), and between the two groups of “partly agree” and “agree”, with a mean difference of 0.38 (*p* < 0.05).

## 4. Discussion

This study—as a first of its kind, to our knowledge—was conducted to obtain a deeper understanding of the documentation technology-related stress and resource situation of German physicians working in neurosurgery and vascular surgery clinics. A main goal within the study framework was to examine potential associations among relevant health- and work-related outcomes as well as to capture the possible need for preventive measures. The study further provided important insights and further details regarding the specific characteristics of technology-associated stressors and contributed to the assessment of needs and special requirements for stress-preventive measures.

### 4.1. Perceived Usability, Perceived Ease of Use, and Technostress

The surveyed physicians, on average, accepted the utilized documentation technologies, with the results of the construct of perceived usability showing that most physicians either slightly agreed or had a neutral view on the technology’s usability. However, the mean of the construct of perceived ease of use was slightly lower, corresponding to a slightly less positive view on the ease of use of the respective technologies, which consequently also indicates that the physicians might also experience difficulties when utilizing digital technologies for medical documentation. These results correspond to the general findings on the aspect of the perception of technologies or on potential barriers towards the use of technologies. In a cross-sectional study by Tajirian et al. (2020), for example, the research team showed that even though 51.1% of the queried physicians believed in an improvement in communication quality and 38.6% believed in an improvement in the quality of patient care through the utilization of EHRs within their department, a share of 62.5% of the physicians still stated that their work with EHRs added to their daily level of frustration, consequently corresponding to a lower perceived ease of use as well [64]. In addition, in a systematic literature review by Boonstra et al. (2010), the authors found that EMRs were often perceived as complex and lacking customizability and were, therefore, considered as difficult to use [78]. Another reason for a lower perceived ease of use might also be a general lack of necessary skills of the physicians or other medical staff to use the respective technology or a consequence of the unmet need for further training or technical support [78]. As presented, the surveyed physicians, on average, reported medium technostress levels, with the highest levels for the techno-stressor of techno-overload, corresponding to a general feeling of the users of being forced by the technologies to work faster and/or longer [30]. These findings mirror the results of other recent studies in the medical sector and among physicians with especially high techno-overload levels measured as well [38,79,80]. The subsequent in-depth correlation and multiple regression analyses further showed moderately negative significant correlations among the factors of perceived usefulness, perceived ease of use, and technostress and showed that the technostress levels could also be explained, to a certain degree (25%), by the two mentioned independent variables, meaning that lower ratings of the subjectively perceived usefulness and ease of use of the technologies led to higher technostress levels and vice versa. This result again matches with recent findings, such as those in the study by Heponiemi et al. (2019), who also showed that high ratings of the user-friendliness of technologies as well as the perceived benefits were associated with lower digital stress levels [81]. Gaube et al. (2019) also proved a certain explanation for higher techno-overload scores through the perceived usability (16% of the variance of techno-overload) [62]. In the systematic literature review by Yan et al. (2021), the authors even found a significant direct and negative association of user-friendliness of EHRs and the burnout risk of the queried healthcare providers, even though these findings were not overall consistent [56]. In a general context, there is already sufficient evidence that the perception of technologies (here, instrumentalized by usefulness and reliability), together with the influence of the techno-stressors, is essential for the evaluation of potentially stressful technology-related situations and conditions. Depending on how this turns out, a situation might either be perceived as challenging without further negative consequences (“techno-eustress”) or as stressful (“techno-distress”). Here, higher values for the usefulness and reliability of technologies were associated with more positive feelings or techno-eustress [59]. In summary, we can accept Hypothesis H_1_, emphasizing the importance of the acceptance of new technologies by the physicians to prevent technostress and emphasizing the importance of preventive measures, which starts with improving the acceptance of the technologies or with the factors that condition technology acceptance.

### 4.2. Specification of Digital Documentation Technology-Associated Stressors and Resources

When being asked about hindering factors or stressors associated with the usage of digital documentation technologies or EHRs, the physicians mentioned, for example, regularly occurring technical failures, a general lack of practicability and a lack of adaptation of the technologies to daily clinical routines, as well as skepticism and obstacles created by the hospital management or a lack of support from the IT team. In addition, only about one-fifth of the participating physicians reported that care was taken to ensure that the new technology system was stable enough when it was introduced. This means that in most clinics, new technologies were introduced, even though they may not have worked properly. As a result, this can lead to technical failures and very time-consuming duplicate documentation.

These results are in line with other scientific findings, suggesting, for example, technical issues and limitations of the technologies, a lack of technical skills of the physicians, a lack of training, qualification, and support from the IT team (technical), or a lack of time to implement new technologies and use them during daily clinical routines (time) as important factors hindering the acceptance of technologies. Additional factors might also be a lack of belief in the advantages of the technologies (psychological), a lack of support from external partners, colleagues, and management, interferences with the doctor–patient relationship (social), concerns about data security (legal), a lack of incentives, a lack of participation, and a lack of leadership during change processes [78].

Our results further show that most of the queried physicians did not have sufficient resources to balance out the potential negative effects of the documentation technologies, with the descriptive analyses of the respective items showing only medium to low levels of technostress inhibitors or resources. Meanwhile, for the resource of literacy facilitation—corresponding to a provision of necessary trainings, qualifications, and information—moderate levels were still measured, and levels for the resource of involvement facilitation were especially low. As described above, these results also reflect the current scientific findings, indicating a lack of the resource of participation, which corresponds to our utilized construct of involvement facilitation [78]. According to Borle et al. (2021), and against the background of the JD-R model, having sufficient influential resources at one’s disposal, however, is very important to mitigate adverse digital stress outcomes [43,82]. Stadin et al. (2020) identified further important resources in addition, which he divided into the categories of “culture, norms and social support”, including co-worker support, for example, “individual resources”, such as digital literacy or learning by doing, and “organizational resources”, which includes good IT support, functioning back-up routines, or administrative support [58]. In the systematic literature review by Rahal et al. (2021), the authors further identified the high-quality and sufficient provision of training, the availability of technology vendors in the case of questions or problems, social support through coaching and peer monitoring, transparency, availability and sharing of information, financial incentives, and adequate technical support as important resources [83]. According to Ludwick et al. (2010), in-house technical support provision is especially essential [84]. Bregenzer et al. (2021) further stressed the importance of leadership and identified a health-promoting leadership style as another important resource [85]. Furthermore, in addition to the technostress inhibitors or resources, coping strategies and the type of coping also play important roles in the interplay of stressors and outcomes and might also mitigate the expression of adverse mental health outcomes [82,86,87].

### 4.3. Status of Implementation and Perception of Preventive Measures

Unsurprisingly, when asking the physicians about their perception of already implemented preventive measures in their clinics, the measures and strategies focusing on a reduction of the above-described stressors as well as focusing on strengthening the above-mentioned relevant resources were the ones described as the most beneficial. The involvement and consultation of the physicians at each step of the selection and implementation process of new technologies, as well as realistically planned technology rollouts and an adaption of the usage purpose of the technologies to daily clinical needs were especially highlighted. As additionally beneficial, the participants described high-quality system maintenance services as well as the adaption of preventive measures, such as qualifications and trainings, to the clinical routines. However, technostress-preventing measures, so far, have only rarely been implemented in clinics, with the physicians, on average, reporting only low to medium levels of implementation. This result is also in line with the findings from a recent German study by the Marburger Bund (2023), which showed that in most of the German hospitals (62%), preventive measures to reduce the digital stress of the employees, such as special IT trainings, are not offered on a regular basis yet [88]. As the most frequently implemented preventive measures in the clinics of the queried physicians, qualifications and a provision of additional information were mentioned. In addition, satisfaction with the already implemented preventive measures also varied a lot. Even though the majority of queried physicians were satisfied with the measures, only 5.3% were very satisfied, and a large share of the participants (30%, n = 114) were not satisfied or not satisfied at all. Consequently, the physicians wished for further preventive measures, such as involvement in the decision-making processes on a regular basis, a provision of additional comprehensive information about the technologies, additional qualifications and training adapted to the clinical routines to ensure physician participation, and simplification of the training contents and information. Lastly, the participants suggested studying and learning from best-practice examples from other clinics. In doing so, they hoped to reduce the additional workload and unnecessary restructuring and adjustments resulting from inadequate planning. At the moment, there are no systematic intervention studies measuring the satisfaction with technostress-preventing measures, and specifically, measuring their quality and potential effects on reducing adverse mental health outcomes, such as burnout, in the medical field, and only very few that have tested or evaluated preventive measures or interventions among other target groups and branches [86]. Some studies carried out in the medical sector, however, have already assessed satisfaction with technology trainings and support programs to some extent, with the main goal of identifying potential barriers to the usage of EHRs [83]. Some of these studies, for example, have pointed to poor-quality and too short technology vendor trainings, as well as to mediation of the training content, which was perceived as far from the reality of clinical routines, and therefore, not meeting the needs of the medical staff, as the sources of dissatisfaction with interventions [83,89,90]. Other studies, however, have shown the positive effects of support programs involving coaching and peer mentoring or change management techniques on the usage of EHRs by physicians [83,89,91]. Further research on the quality and effects of preventive measures is still highly indicated and an important starting point for their successful implementation in hospitals.

### 4.4. Relationships among Techno-Stressors and Technostress inhibitors and Burnout Symptoms, General Health Status, and Job Satisfaction

In analyzing the relationship of the techno-stressors and technostress inhibitors or resources with the different health- and work-related outcomes in detail, our results show small a positive correlation between the expression of techno-stressors and the expression of subjectively perceived burnout symptoms, a small negative correlation between the technostress level and the subjectively perceived general health status, as well as a certain explanation, albeit small, of the prevalence of burnout symptoms by the level of technostress. These results are in line with the recent literature showing, for example, in a Canadian study conducted in the medical sector, that 69.3% of the queried physicians also perceived that EHR-associated stressors contributed to their burnout symptoms [63]. Other recent quantitative studies in the medical field have also shown significant positive correlations between technostress levels and the burnout variable and a good explanation of the outcome of burnout based on the level of technostress [62,64], as well as significant negative correlations between the variable of technostress and general health status, which is, however, often not as strong in comparison [64].

The correlation between technostress and the outcome of job satisfaction was not significant, consequently meaning that Hypothesis H_2_ was not fulfilled completely. This is in contrast to recent comparable studies indicating significant negative correlations between the different techno-stressors or the general technostress levels and the outcome of job satisfaction [61,64]. A reason for this deviating result might, for example, be the influence of mediator variables [79]. In a Korean study conducted among nurses, the authors, for example, identified workplace learning (with an explanatory power of 27%) and employee’s self-efficacy (with an explanatory power of 10%) as relevant (partial) mediating variables (ibid.). In addition, we detected a small positive correlation between the technostress inhibitors or resources and the level of job satisfaction. Consequently, this finding might also support the assumption that technostress inhibitors or resources also function as another mediator variable. In the study by Hang et al. (2021), the authors, for example, showed that technostress inhibitors had a mediating effect on technostress levels and the employees’ well-being [39]. Another recent German study, which investigated the effects of telepressure, as an ICT demand, on several outcomes, also showed a significant moderating effect between technostress creators and technostress inhibitors for the outcomes of work engagement and job satisfaction [92]. Another Pakistani study further identified self-efficacy and training as significant moderator variables on the relationship between technostress and the outcome of employee performance, whereas high levels of self-efficacy and high levels of training could mitigate the negative effects of perceived technostress on employee performance [93]. The other analyses investigating assumed correlations between the variables of technostress inhibitors and the outcomes of burnout symptoms and general health status were, however, not significant, meaning that Hypothesis H_3_ cannot be accepted completely either. In general, the queried physicians experienced burnout symptoms only rarely, were mostly satisfied with their job, and on average, described their health status as good. As studies measuring the technostress and burnout levels among physicians working in neurosurgery or vascular surgery have not been conducted yet, comparisons of these results are difficult. However, the study results by Gardner et al. (2019), who measured and compared the technostress levels and persistence of burnout symptoms in different medical disciplines, can be consulted, showing indeed only low expressions of burnout symptoms among physicians working in general surgery (16.7% of participants) or orthopedic surgery (23.9% of participants) [63]. A reason for the overall low levels of adverse mental health outcomes or high expressions of the other positive outcomes might be a potential influence of not-included mediator variables, as described previously, but might also lie in the underrepresentation of certain subgroups of study participants among the queried physicians, which is described in more detail in Section 4.7.

### 4.5. Age and Gender-Related Differences in Technostress Levels and Burnout Symptoms

In analyzing the potential differences in the two outcomes of technostress and burnout symptoms between the genders and the different age groups, we found significant differences in burnout symptoms between men and women, with women experiencing burnout symptoms to a higher degree than men. This result is consistent with recent studies measuring burnout either in the context of the implementation of digital technologies or without the context of digital technologies and suggesting that female physicians have higher odds of experiencing burnout symptoms [53,55,69,94]. The results of the potential differences between the genders regarding (technology-related) stress, however, have not always been consistent. Galaiya et al. (2020), for example, showed that female gender was associated with higher stress rates [69]. However, in this study, the authors measured stress only in a general context, and as such, did not measure the influence on technostress levels specifically. Furthermore, technostress levels and burnout symptoms did not differ significantly among the different age groups, meaning that Hypothesis H_4_ was fulfilled only in part. These results also mirror the current scientific findings showing, indeed, that the results regarding the associations of age and burnout with (techno-)stress are, at the moment, inconsistent [30,32,63,65,69,94]. While Galaiya et al. (2020) suggested that a younger age of surgeons could be associated with higher stress levels—while only measuring stress levels in general and not specifically technostress levels—[69], Gabr et al. (2021) and Golz et al. (2021) showed the contrary [32,65]. Another quantitative study and another meta-analysis could also not confirm their hypothesis on the association of age with (techno-)stress [63] and on the hypothesized association of younger age with burnout and stress [94].

### 4.6. Influence of Preventive Measures on Technostress

Even though our results only show moderate technostress levels and no strong negative expressions of the above-described outcomes, the implementation of measures aimed at preventing technostress, however, is still very important to prevent potential negative effects resulting from the utilization of technologies already at an early stage, especially given that relevant resources were not available to a sufficient degree in this sample. In addition, the conducted difference test showed significant differences in technostress levels depending on the degree of implementation of preventive measures, with the participants whose employers have already implemented a lot of measures and the participants whose employers have not at all differing the most significantly, consequently showing different levels of technostress with higher levels for the group of physicians with a low degree of implementation of preventive measures. Given these results, we consequently can accept Hypothesis H_5_.

These results are also reinforced by a recent study that tested a blended online intervention to reduce digital transformation stress and to improve digital transformation attitudes, emerging due to the increased use of digital technologies during the COVID-19 pandemic. In the study, the authors proved that among the employees who suffered from high digital transformation stress levels before the intervention and who were actively involved to participate reported lower stress levels after the intervention [95]. Furthermore, they showed a lower level of negative emotions towards digital transformations after the intervention (ibid.).

The importance of the implementation of measures to prevent adverse health outcomes was also proven in a systematic literature review on the effects of different interventions by Craig et al. (2021), who showed that in 68% of the presented 38 studies, digitization-associated burnout symptoms could be improved through the integration of different preventive measures [96]. As indicated above, further research on the effectiveness of technostress-preventing measures is still needed. A suggestion for the first measures that might be implemented on different levels shall is presented in the next chapter.

### 4.7. Strengths and Limitations

The strengths of this study include, among others, the fact that we used various validated and well-established scales, such as the TAM model, the technostress scale by Ragu-Nathan et al. (2008), and the different scales from the COPSOQ, ensuring the quality of the measurements, and consequently, of our results. Another strength lies in the design of our recruitment strategy, which was based on official member overview lists of the specific surgical societies and, as such, ensured a complete selection and contact with the clinics. However, certain limitations of our study also need to be addressed. In general, at this point it is first important to draw attention to the fact that most of the surveyed physicians were head physicians or senior physicians (79.8%; n = 114), while only 13.2% of the physicians were assistant physicians. This might have posed a bias in our results, as assistant physicians presumably spend more time utilizing the documentation technologies and might be more stressed by it as they, in general, are also known to experience more stress, while at the same time, have fewer resources at their disposal [97]. Due to the small number of study participants or due to the underrepresentation of assistant physicians and specialist physicians within the study sample, a conduction of tests analyzing potential differences in the technostress levels among the different groups was not possible. In addition, female physicians were underrepresented in this study, which might have posed another bias too. The survey recruitment strategy should, therefore, be improved to avoid the underrepresentation of certain participant groups, such as assistant physicians, specialist physicians, or female physicians, as in the present study. It might, for example, be an idea to focus on the creation of incentives for the respective subgroups to participate in studies such as this one.

Furthermore, it was noticed by the research team that the term “preventive measures” might not have been understood by all study participants in the same way or not always as intended by the researchers, which led to an unavoidable exclusion of some of the participants’ answers and which might point out a need to improve some of the introduction texts and item explanations of the questionnaire for future use and/or to improve communication about the aim of the study beforehand. In general, it should also be pointed out that due to the small sample size of our study and due to the underrepresentation of certain groups of participants, as already described above, the representativeness, and consequently, the generalizability of our study results might be limited. A higher number of study participants should, therefore, be targeted in future studies on the topic. However, most of our results are in line with the recent findings from international and German studies, as described above, and the results from our qualitative data analysis further provide additional valuable insights into the digital stress experience of the queried target group, as well as further details about the requirements for the design and implementation of stress-preventing measures, therefore providing an important contribution to the current research. Regarding the utilized variables, it might be considered in future research to include additional influencing or mediating factors on technostress and the different outcomes, such as digital competence, self-efficacy, or training. In a more general context, additional psychological stress factors characterizing the working environment of physicians, such as daily workloads, time pressure, emotional demands, high task complexity, or small decision-making scopes, might also have biased our results [50]. Due to the thematic limitation of our study, we had to limit our independent variables. However, since the above-listed stress factors represent additional important influencing factors on physician burnout, they should be included in future research. Furthermore, the associations of individual characteristics of the study participants, such as gender and age, with different outcome variables should be analyzed in more depth, especially given the inconsistent findings of other studies at the moment. In general, due to the cross-sectional study design of the underlying survey, statements about the causality of, e.g., technostress and adverse mental health outcomes, as well as other associations among the variables, of course, cannot be made. We, therefore, recommend to extend surveys like the present one in the form of a longitudinal study design with follow-up surveys. In addition, the present findings might also be complemented and extended by additional information gained through additional research methods, such as qualitative interviews or focus groups, to deepen the level of information content.

### 4.8. Implications for Further Research and Practice

#### 4.8.1. Recommendations for Future Research

First, as already suggested above, the sample size of future quantitative studies on the topic should be increased to improve the generalizability of the results. In this context, particular attention should be paid to the design of the recruitment strategies, potential factors hindering survey participation, and factors increasing the motivation to participate to ensure evenly distributed sizes of the subgroups among the target group, and as such, to increase diversity within the target group [98]. In the present study, especially assistant physicians, specialist physicians, and female physicians were underrepresented. Future research should consequently be aimed at increasing the number of named physician subgroups because, although some studies have already suggested a higher exposure of participants with the above-named characteristics to technology-induced stress as well as higher risk for developing burnout, the findings are not consistent yet, indicating further need for research [32,53,55,63,65,69,94,97]. Furthermore, comparable studies should also be conducted in a longitudinal design to be able to analyze the development of technostress levels and possible changes over a longer period of time. In addition, further research is needed regarding potential additional influencing variables with an impact on the technostress levels of physicians working in the field of neurosurgery or vascular surgery, as knowledge about them is still very new. In this context, further mediating variables should be included to further improve the understanding of the relationships among technostress creators and resources and different outcomes [39,80,92,93]. Lastly, there is still a lack of necessary information on effective preventive measures and experience with their implementation. Further research is, therefore, especially needed to improve the knowledge about how preventive measures need to be designed to effectively prevent technostress. To improve this research gap, field-based intervention studies examining the quality of and the satisfaction with different preventive measures are highly indicated [86].

#### 4.8.2. Practical Implications

As described above, several studies have already examined the effects of different measures aimed at preventing digital stress and/or adverse mental health outcomes associated with the utilization of digital technologies and/or aimed at improving job satisfaction. As described in the previous chapter, there is still a lack of information about which preventive measures are suitable for effectively preventing technostress in the group of physicians working in neurosurgery and vascular surgery clinics at the moment, and intervention studies need to be conducted to gain evidence-based knowledge about the effectiveness of preventive measures. Based on the findings of this study, and based on the most recent findings from other studies among different target groups, the following preventive measures however can still be recommended to be implemented on a technological, organizational, or individual level to both reduce stressors and strengthen resources (see Table 12 below) [58,95,99]:

It has to be added that, according to Craig et al. (2021), the effect of preventive measures on the technological level seemed to be weakest in comparison with other intervention types in the medical sector among physicians [96]. However, interventions aimed at adapting and improving the technologies or usage processes to the special requirements in the medical field—especially when including the wishes of the physicians—in combination with special user trainings proved to be effective in reducing burnout symptoms (ibid.).

## 5. Conclusions

This study gives a first overview about the persistence of techno-stressors, technostress inhibitors, and technostress levels, as well as possible influences on relevant health- and work-related outcomes within a group of physicians working in neurological and vascular surgery clinics in Germany. As a main conclusion, this study shows that knowledge about the potential causes of stress from the utilization of digital technologies is not yet widespread in clinics and that stress-preventing measures have only very rarely been implemented so far. As described previously, even very essential preventive measures, such as special IT trainings offered on a regular basis, are sometimes not even implemented. This is especially problematic, as physicians in this study, on average, showed moderate technostress levels, while at the same time, only have few resources. In general, it is also already known that physicians are exposed to a complex composition of stressors at their workplace and are at risk of experiencing negative stress outcomes, such as burnout. Against the background of the most recent scientific findings and the findings of the present study, a strategic and quality-guided implementation of measures to effectively prevent digital stress should go hand in hand with the implementation of new digital technologies in order to shape digitization processes as stress-free as possible, and thus, to be able to profit from the whole potential that the digitization of hospitals has to offer. In addition to a further general extension of the research on the present topic to further improve the knowledge on the interplay of different stressors, resources, and outcomes, through additional research methods, future research should also especially focus on intervention studies to gain further knowledge about the effectiveness of preventive measures.

## Figures and Tables

**Table 1:** Characteristics of study population and hospitals (n = 114).

Characteristic	Frequency (n)	Percentage (%)
Gender		
Male	78	68.4
Female	36	31.6
Age		
20–29 years	3	2.6
30–39 years	27	23.7
40–49 years	27	23.7
50–59 years	37	32.5
60 years and older	20	17.5
Surgical department		
Neurosurgery	52	45.6
Vascular surgery	62	54.4
Job position		
Assistant physician	15	13.2
Specialist physician	8	7
Senior physician	53	46.5
Head physician	38	33.3
Extent of current employment		
Working full time (≥35 h/week)	104	91.2
Working part time (15–34 h/week)	10	8.8
Duration of employment with employer		
<1 years	9	7.9
1–<2 years	7	6.1
2–<3 years	9	7.9
3–<4 years	2	1.8
≥4 years	87	76.3
Overall clinical experience		
<5 years	4	3.5
5–<10 years	17	14.9
10–<15 years	16	14
15–<20 years	15	13.2
20–<25 years	22	19.3
≥25 years	40	35.1
Hospital sponsorship		
Commercial sponsor (profit-oriented)	20	17.5
Public sponsor	65	57
Independent sponsor (non-profit, charity, church)	29	25.4
Regional structure of employer		
Municipal	71	62.3
Provincial	41	36
Rural	2	1.8

**Table 2:** Usage frequency and usage duration of documentation technologies (n = 114).

Characteristic	Frequency (n)	Percentage (%)
Usage frequency		
Daily usage	113	99.1
Usage several times per week	1	0.9
Usage duration (estimated per day)		
<1 h	2	1.8
1–<2 h	28	24.6
2–<3 h	44	38.6
3–<4 h	22	19.3
4–<5 h	11	9.6
5 h or more	7	6.1

**Table 3:** Expressions of techno-stressors within sample (n = 114).

Techno-Stressors	Mean (M)	Standard Deviation (SD)
Techno-overload	3.09	0.86
Techno-complexity	2.25	0.87
Techno-uncertainty	3.03	0.88
Overall expression of techno-stressors	2.79	0.65

**Table 4:** Expression of technostress inhibitors within sample (n = 114).

Technostress inhibitors/Resources	Mean (M)	Standard Deviation (SD)
Literacy facilitation	3.10	1.05
Involvement facilitation	1.91	0.90
Overall expression of resources	2.51	0.85

**Table 5:** Expression of burnout symptoms, job satisfaction, and subjectively perceived general health status within sample (n = 114).

Variable Expression	Mean (M)	Standard Deviation (SD)
Burnout symptoms	2.92	0.97
General health status	7.62	1.84
Job satisfaction	4.05	0.63

**Table 6:** Qualitative evaluation of items with free-text answers in the form of a category system.

Items/Main Categories	Subcategories
Stress-inducing aspects	▪ Technical problems/immature technical solutions; ▪ Lack of support from IT team/deficient planning; ▪ Insufficient usage adaption to everyday clinical practice; ▪ Resistance/skepsis from hospital management; ▪ Dependance on technologies.
Benefits of implemented preventive measures	▪ Integration and consultation of clinical personnel during technology selection processes; ▪ Realistic daily routine of clinics adapting to technology rollouts; ▪ Adaption of preventive measures to needs of clinic personnel; ▪ Provision of information and transparency during technology introduction processes; ▪ High quality of system maintenance service; ▪ No benefits of preventive measures.
Criticism regarding implemented preventive measures	▪ Lack of involvement in technology selection and implementation processes; ▪ No existing/too short trainings on technologies; ▪ No time for trainings/lack of adaption of trainings to clinical daily routines; ▪ No support after technology introduction; ▪ No effects/use of trainings.
Wish for additional preventive measures	▪ Involvement in technology selection processes; ▪ More comprehensive information, training and support from IT team; ▪ Simplification of training contents; ▪ Adaption of training courses to everyday clinical working routines; ▪ Regular provision of information on possible improvements/updates (e.g., best practice/learning from other clinics).

**Table 7:** Presentation of Pearson correlation coefficients for technostress, PU and PEOU (n = 114).

				Overall Expression of Techno-Stressors	Perceived Usefulness (PU)	Perceived Ease of Use (PEOU)
Overall expression of techno-stressors	Pearson correlation			1	−0.372 **	−0.452 **
	Sig. (2-tailed)				0.000	0.000
	N			114	114	114
	Bootstrap ^1^	Bias		0	−0.005	0.000
		Std. Error		0	0.082	0.085
		95% Confidence Interval	Lower		−0.529	−0.610
			Upper		−0.225	−0.275

** Correlation is significant at the 0.01 level (2-tailed). ^1^ Unless otherwise noted, bootstrap results are based on 1000 bootstrap samples.

**Table 8:** Multiple regression analysis of variables of PU, PEOU, and technostress (n = 114).

Predictors	b ^a^	*SE* ^a^	t	*p*
Perceived usefulness (PU)	−0.183 (−0.318, −0.048)	0.068	−2.684	<0.05
Perceived ease of use (PEOU)	0.311 (−0.478, −0.144)	0.084	−3.689	<0.05

Notation. R^2^ = 0.252 (n = 114, *p* < 0.001). ^a^ Confidence intervals und standard errors per BCa bootstrapping with 1000 BCa samples.

**Table 9:** Presentation of Pearson correlation coefficients for technostress and outcomes (n = 114).

				Overall Expression of Techno-Stressors	Burnout Symptoms	General Health Status	Job Satisfaction
Overall expression of techno-stressors	Pearson correlation			1	0.214 *	−0.194 *	−1.82
	Sig. (2-tailed)				0.022	0.038	0.053
	N			114	114	114	114
	Bootstrap ^1^	Bias		0	−0.003	−0.005	−0.004
		Std. Error		0	0.102	0.120	0.115
		95% Confidence Interval	Lower		0.008	−0.411	−0.400
			Upper		0.395	0.027	0.041

* Correlation is significant at the 0.05 level (2-tailed). ^1^ Unless otherwise noted, bootstrap results are based on 1000 bootstrap samples.

**Table 10:** Multiple regression analyses of the variables techno-overload, -complexity, -uncertainty, and the outcome variables of burnout symptoms and job satisfaction (n = 114).

Predictors	b ^a^	*SE* ^a^	t	*p*
	Outcome of Burnout Symptoms			
Techno-overload	0.442 (0.242, 0.641)	0.101	4.382	<0.001
Techno-complexity	−0.590	0.096	−0.613	>0.05
Techno-uncertainty	−0.110	0.083	−1.316	>0.05
	Notation. R^2^ = 0.125 (n = 114, *p* < 0.001).			
	Outcome of Job Satisfaction			
Techno-overload	−0.163 (-0.297, −0.029)	0.068	−2.402	<0.05
Techno-complexity	−0.068	0.064	−1.052	>0.05
Techno-uncertainty	0.088	0.056	1.567	>0.05
	Notation. R^2^ = 0.068 (n = 114, *p* < 0.05).			

^a^ Confidence intervals und standard errors per BCa bootstrapping with 1000 BCa samples.

**Table 11:** Presentation of Pearson correlation coefficients for technostress inhibitors and burnout symptoms, general health status, and job satisfaction (n = 114).

				Overall Expression of Technostress Inhibitors	Burnout Symptoms	General Health Status	Job Satisfaction
Overall expression of technostres inhibitors	Pearson correlation			1	−0.009	0.118	0.190
	Sig. (2-tailed) *				0.924	0.211	0.043
	N			114	114	114	114
	Bootstrap ^1^	Bias		0	−0.004	−0.005	0.001
		Std. Error		0	0.091	0.097	0.088
		95% Confidence Interval	Lower		−0.196	−0.069	0.009
			Upper		0.167	0.298	0.355

* Correlation is significant at the 0.05 level (2-tailed). ^1^ Unless otherwise noted, bootstrap results are based on 1000 bootstrap samples.

**Table 12:** Overview of preventive measures reducing techno-stressors and strengthening resources.

Level of Measure Implementation	Reducing Stressors	Strengthening Resources
Technological	▪ Adaption of technologies to requirements of working place, and involvement and empowerment of employees; ▪ Adaption of technologies to needs of employees; ▪ Release management (effective and adapted planning of implementation processes to individual needs); ▪ Implementation of additional technologies to support the task completion (e.g., reminder functions or addition of decision support systems to EHRs); ▪ Selection of intuitive and easy-to-use technology.	▪ Use of gamification (technologies with playful and/or rewarding elements).
Organizational	▪ Development of a data protection concept; ▪ Implementation of a company agreement (definition of goals and framework of the technology use by both management and employees); ▪ Definition of internal norms and standards for the use of the technologies (e.g., through workshops) to reduce excessive time consumption by technologies; ▪ Implementation of a change management (offering of up-to-date and timely information and training); ▪ Implementation of an IT helpdesk to offer fast support; ▪ Improvement of internal workflows and/or quality improvement initiatives addressing physician’s concerns.	▪ Development of a cooperative digitization culture and of a co-worker support structure; ▪ Development of a communication guideline (development of rules for open and transparent communication); ▪ Organizational restructuring and expansion of personal resources/teams.
Individual	▪ Special training (e.g., to learn how to mono-task instead of multi-task); ▪ Establish platforms to exchange experiences and best practice examples.	▪ Offering of IT trainings (at least when introducing new technologies); ▪ Offering of trainings to increase awareness of digital stress and to learn about coping strategies; ▪ Offering of courses/measures to balance out digital stress (e.g., sport courses, mindfulness courses); ▪ Offering of trainings/workshops for/with managers; ▪ Mentoring for digitization topics (to help with questions/offer information).

## Data Availability

The data analyzed during the current study are not publicly available due to German national data protection regulation. They are available upon individual request from the corresponding author.

## Abbreviations

EHR: electronic health record, EMRAM: Electronic Medical Record Adoption Model, ICT: information and communication technologies, PDA: personal digital assistant, KHZG: Krankenhauszukunftsgesetz (Engl. Hospital Future Law), JD-R-model: job demands–resources model, TAM: technology acceptance model, PU: perceived usefulness, PEOU: perceived ease of use, HIMSS: Healthcare Information and Management Systems Society.
